# Na^+^ Micro-Current Value Detection as a New Modality for Identification of Benign and Malignant Disease in Surgery

**DOI:** 10.1038/srep24937

**Published:** 2016-04-22

**Authors:** Xu-Feng Zhang, Zhi-Da Long, Xue-Min Liu, Feng Ma, Qiang Li, Yi Lv

**Affiliations:** 1 Department of Hepatobiliary Surgery, the First Affiliated Hospital Xi’an Jiaotong University, Xi’an 710061, China; 2 Institute of Advanced Surgical Technology and Engineering, the First Affiliated Hospital Xi’an Jiaotong University, Xi’an 710061, China; 3 Shaanxi Provincial Regenerative Medicine and Surgical Engineering Research Center. Xi’an 710061, China; 4 Jingzhou Hospital, Tongji Medical College, Huazhong University of Science and Technology, Jingzhou 434020, China; 5 Jiangsu Kunshan Bokang Medical Technology Co. Ltd., Kunshan 215300,China

## Abstract

Increase of intracellular positive ions (mainly Na^+^) indicates greater possibility of cell malignancy. The present study investigated the correlation between the Na^+^ micro-current value (MCV) and tissue characteristics (normal, benign or malignant). 346 tissue samples have been detected within 30 min after surgical isolation by Na^+^ detector. MCV in 102 malignant tumor was significantly higher than that in benign/borderline tumor or normal tissue (33.3 ± 8.9 μA vs. 24.4 ± 8.6 μA and 14.0 ± 4.0 μA, *p* < 0.001, respectively). MCV in malignant tumor parenchyma was significantly higher than that in the paired paracanceroustissue, normal tissue and surgical margin tissue (33.3 ± 8.9 μA vs. 18.9 ± 4.1, 14.2 ± 4.0 or 15.2 ± 3.3, *p* < 0.001, res*p*ectively). However, the coincidence rate between Na^+^ detector and pathological examination was different in tissues from different organs or systems, which was high in pancreas, bile duct system, gastrointestinal system, esophagus, breasts, lungs, nose & throat and thyroids, but poor in urinary tissue. The overall coincidence rate was 83.1% (108/130) between Na^+^ detector and pathological examination. The sensitivity and specificity of correct diagnosis by Na^+^ detector was 83.3% (70/84) and 82.6% (38/46), respectively. This new modality may have diagnostic potential in complementing frozen examination in differentiating malignant tumor from benign or normal tissue, justifying tumor metastatic scope and confirming surgical margin.

Surgical resection remains the optimal treatment with potential eradication of most solid tumor. However, it is frequently occurred intraoperatively to surgeons to differentiate benign from malignant lesions, evaluate the spread scope of tumors, justify the positive lymph nodes involved and determine the surgical margin, which are all critical in surgical plan. Traditionally, fast biopsy examination, such as frozen section examination of fresh tissue, is performed. However, frozen sections are impossible to achieve the accuracy of the quality of paraffin sections due to limitation of specimen collection, short time of production and dyeing of the slices. Therefore, accuracy of fast frozen biopsy ranges from 88.9% to 98.9%, which implies that the misdiagnosing rate is about 5%^1^,^2^. And intraoperative fast frozen biopsy always takes more than 30 min before diagnosis, thus prolongs operation time. In some cases, it is difficult to differentiate malignant from benign lesions by frozen biopsy, and further paraffin section examination is always needed.

Since 1960s, it was found that cell membrane potential depolarized significantly during malignant transformation process of normal cells^3^. Accordingly, Cone *et al.* propounded the theory that changes of membrane potential are correlated with cell mitotic activity^4^. Previous studies have suggested that rapidly proliferating and transformed cells have electrically depolarized cell membranes compared with normal cells^5^,^6^. And depolarization accompanies with cation influx, resulting in increase of intracellular cation concentration. Further studies revealed that elevated intracellular cation concentrationwas positively correlated with cell division rate^5^,^7^,^8^. And increase of intracellular positive ions indicated malignancy of tissue^5^,^7^,^8^,^9^. In tumor cells, it was mainly Na^+^ increased significantly than nontumor cells in concentration, but K^+^ remains relatively stable in concentration^10^,^11^. It was reported that the percentage of Na^+^ concentration in normal tissue, benign or borderline tumor and malignant tumor cells were 0.05%, 0.05–0.07% and 0.15–2%^10^. It was also found that the number of cations was 138–196 mmol/L in every kilogram of dry normal tissues, but reached 451 mmol/L in malignant tissues^7^,^12^. Dr. Laszlo Ungar and his colleagues had developed a micro current sensor (LEC-03 Cancer Detector) to examine the cation concentration in cells and tissues, and used it for cancer diagnosis^13^,^14^.

By applying Na^+^ detector, we have tested the Na^+^ micro-current value (MCV) in 346 fresh tissues from 139 patients intraoperatively. The Na^+^ MCV in tumors and adjacent tissues or normal tissues were compared, and the proposed diagnosis by Na^+^ detector was also evaluate with final pathological examination.

## Results

Totally, 139 patients undergoing tumor resection have been enrolled. Amongst, there were 100 patients with malignant tumors, 35 with benign tumors and 4 with borderline tumors after pathological examination. 346 tissue samples have been used for Na^+^ detection. Amongst, there were 102 cases of malignant tumor parenchyma tissue, and 59 paired cases of paracancerous tissue, 41 cases of surgical margin tissue and 76 cases of normal tissue. There were also 43 cases of benign tumors and paired 20 cases of normal tissue, and 4 cases of borderline tumor (Table 1).

### Comparison of Na^+^ MCV between malignant and benign tissue

As shown in Table 2, MCV in 102 malignant tumor was significantly higher than that in benign/borderline tumor or normal tissue (33.3 ± 8.9 μA vs. 24.4 ± 8.6 μA or 14.0 ± 4.0 μA, *p* < 0.001, respectively). Moreover, MCV in malignant tumor parenchyma was significantly higher than that in the 59 paired cases of paracancerous tissue, 76 paired cases of normal tissue and 41 paired surgical margin tissue (Table 3, *p* < 0.001, respectively). MCV in paracancerous tissue was also higher than that in the paired normal tissue (Table 3, *p* < 0.001). It was interesting that MCV in 43 benign tumor was significantly higher than that in the 19 paired normal tissue (24.3 ± 9.0 μA vs. 13.5 ± 3.9 μA, *p* < 0.001), which was, however, not statistically different from MCV in 4 cases of borderline tumor (24.3 ± 9.0 μA vs. 25.8 ± 3.0 μA, p > 0.05).

### MCV in tissues from different organs or systems

We further analyzed Na^+^ MCV in tissues from different organs or systems. As shown in Table 4, MCV of malignant tissue was significantly higher than that of the paired paracancerous tissue, surgical margin tissue or normal tissue in most organs including liver, pancreas, gallbladder and bile duct, gastrointestinal tract, esophagus, thyroid, nose and throat, breasts and uterus tissue (Table 4, all *p* < 0.05). As analyzed, MCV was higher in malignant tumor compared to benign tumor from thyroid and breasts, respectively (Table 4, both *p* < 0.05). However, MCV in malignant tumor from urinary system was not different from that in the paired paracancerous tissue (Table 4, *p* > 0.05) but still higher than that in the paired normal tissue (Table 4, *p* < 0.05). Therefore, we excluded urinary samples from the study in further analysis including 16 malignant tumors, 3 borderline tumors, 8 paracancerous tissue and 9 normal tissue.

### Comparison of the results between Na^+^ detector and pathological examination

**Figure 1:**
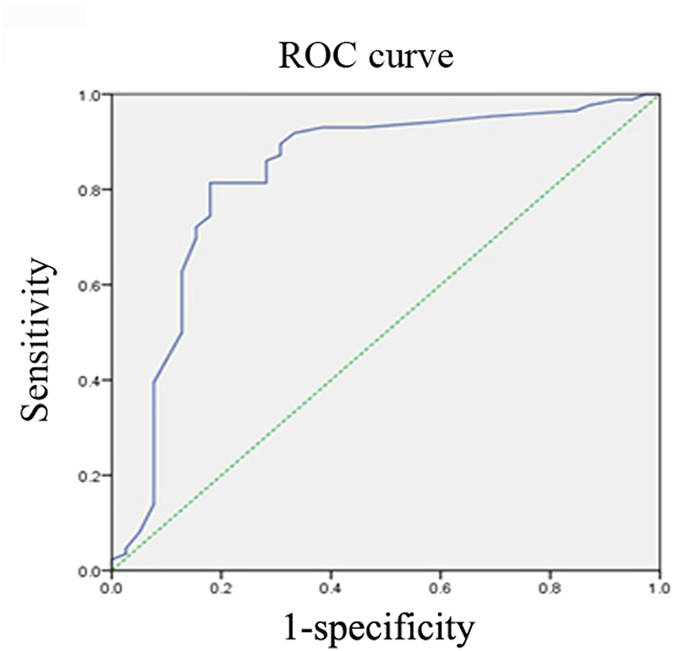
Receiver operating characteristic (ROC) curve for diagnosis of benign and malignant tumors by Na^+^ micro-current value detector. Area under ROC curve was 0.842. When the threshold was 30 μA, the sensitivity and specificity of correct diagnosis were 83.3% and 82.6%, respectively.

After exclusion of urinary tissue, 86 cases of malignant tumor and 44 cases of benign or borderline tumor from 130 patients were diagnosed with pathological examination. According to the criterion of Na^+^ detector, 70 cases of tissues with MCV higher than 30 μA were confirmed as malignant disease, while 38 cases of tissues with MCV lower than 30 μA were diagnosed as benign disease pathologically (Table 5). The overall coincidence rate was 83.1% (108/130) between Na^+^ detector and pathological examination. The sensitivity and specificity of correct diagnosis by Na^+^ detector was 83.3% (70/84) and 82.6% (38/46), respectively (Table 5). Area under ROC curve was 0.842 (Fig. 1). When the diagnostic threshold was 29.5 μA, the maximum Youden’s index was 0.828. The results confirmed that the optimal diagnostic threshold was 29.5 μA, which was consistent with the recommended value by the manufacturer (30 μA).

Noticeably, the compliance rate between Na^+^ detector and pathological examination was slightly different in different organs, which was 50% (4/8) in liver, 91% (10/11) in pancreas, 91% (10/11) in biliary system, 84% (21/25) in gastrointestinal tract, 100% (5/5) in esophagus, 71% (20/25) in thyroid, 75% (4/5) in nose and throat, 92% (13/15) in breasts, 100% (4/4) in lungs, 58.3% (7/12) in uterus, and, 86% (6/7) in lymph nodes, etc.

### Evaluation of Na^+^ MCV in surgical margin tissue

**Figure 2:**
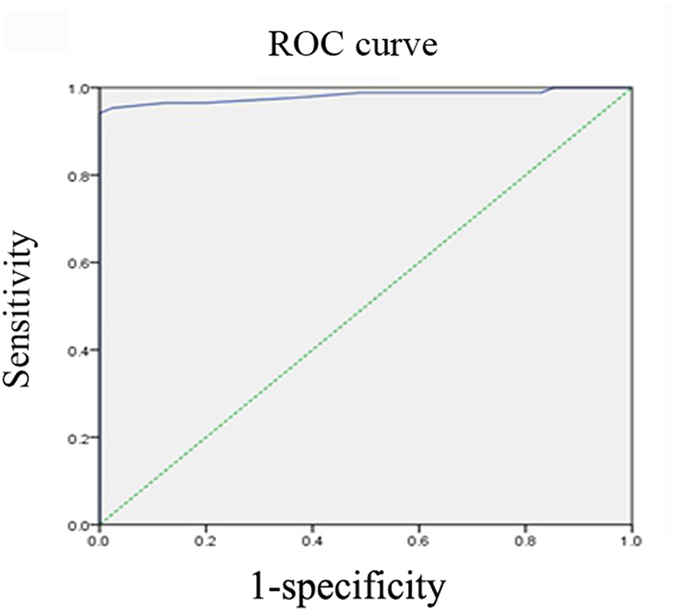
Receiver operating characteristic (ROC) curve for diagnosis of surgical margin by Na^+^ micro-current value detector. The area under the ROC curve was 0.981. When the threshold was 21.5 μA, the sensitivity was 94.2% and the specificity was 100%.

Na^+^ MCV in 86 cases of malignant disease was significantly higher than the paired 41 cases of surgical margin tissue (33.3 ± 8.9 μA vs. 15.2 ± 3.3 μA, *p* < 0.001, Table 3). Interestingly, all the 41 surgical margin tissue had negative value (MCV < 30 μA). However, 14 out of 84 (16.7%, Table 5) malignant tumors showed MCV lower than 30 μA as well, which was defined as false negative rate. Therefore, it might be necessary to find out the optimal diagnostic threshold value to improve the detection sensitivity in surgical margin tissue. A further analysis by ROC curve was performed with 86 cases of malignant tumor and 41 paired surgical margin tissue (Fig. 2). The area under the ROC curve was 0.981 (Fig. 2). The maximum Youden’s index was 0.942 when the diagnostic threshold was 21.5 μA. The sensitivity was 94.2%, while the specificity was 100%.

## Discussion

Our results showed that Na^+^ detector provided highly significant discriminatory information for malignant tumor and non-tumor tissues. The significant difference of MCV was present between malignant tumor and benign/borderline tumor or normal tissue. Moreover, MCV in malignant tissue was dramatically increased compared with paired paracancerous tissue, surgical margin tissue or normal tissue. The gradient increase of Na^+^ MCV from normal tissue, benign/borderline to malignant tumor might verify that cation concentration is negatively indicative of the degree of tissue differentiation. It has been found that membrane potential was significantly depolarized in rapidly dividing tumor cells, slightly depolarized in proliferative somatic cells, but polarized in resting normal cells^4^,^8^,^9^,^11^. Cell membrane depolarization accompanies with cation influx, resulting in increase of intracellular cation concentration and PH value. And increase of intracellular positive ions (mainly Na^+^) indicated malignancy of tissue^5^,^7^,^8^,^9^. Therefore, it is possible to differentiate malignant from benign or normal tissue by Na^+^ MCV. Actually, application of cation detector in diagnosing malignant tumor has been sparsely reported with small number of patients enrolled^15^,^16^,^13^,^14^,^17^. The overall coincidence rate between Na^+^ detector and pathological examination ranged from 86.7% to 97.7% 15,16,13,14,17, which was 83.1% in the present study.

Considering the difference of biological characteristics, we evaluated Na^+^ MCV in malignant tissue and the paired non-tumor tissue from different organs or systems. Consistently, MCV in malignant tumor parenchyma was significantly higher than that in benign tumors, paracancerous tissue, surgical margin tissue or normal tissue in all the organs and tissues we detected. Noticeably, MCV in malignant uterine tumor was not different from that in uterine myoma, but higher than that in the paired paracancerous tissue or normal tissue. Intriguingly, MCV in most malignant tumors of uterus was below 30 μA. Although the reasons remain unknown, the intrinsic ion metabolism of uterine tumor might be the major influential factor.

A 30 μA threshold value has been recommended by the manufacturer. And we also examined that when the threshold was 29.5 μA, we would get the best diagnostic results with sensitivity of 83.3% and specificity of 82.6%. It remains unknown that the coincidence rate between Na^+^ detector and pathological examination was somehow different in tissues from different organs or systems. We found highly diagnostic accuracy of Na^+^ detector in pancreas, bile duct system, gastrointestinal system, esophagus, breasts, lungs, nose & throat and thyroids, moderately in liver, duodenal papilla and uterus. Considering the statistical difference of MCV in 86 malignant tumor and 41 paired surgical margin tissue, the optimal threshold for diagnosis was 21.5 μA by ROC curve. However, by tissue specific analysis, the optimal threshold for diagnosing surgical margin was 25 μA, 26 μA and 24 μA in gastrointestinal tumors, gallbladder/bile duct tumor and pancreatic tumor. Therefore, it is likely that Na^+^ MCV might have different diagnostic potential in different tissue samples. Further analysis might be necessary in confirmation of the tissue specific optimal threshold.

Electrodiagnosis has been practiced clinically mainly in breast lesions, although its cellular/molecular basis remains unknown^18^,^19^,^20^. It has been shown that upregulation of voltage-gated Na^+^ channel in cancer cells was necessary and sufficient for cellular invasion potential^21^,^22^,^23^,^24^. Ion channels and transporters (ICT) are a new class of membrane proteins which are aberrantly expressed in several types of human cancers^22^,^25^,^24^,^26^. Different types of ICT have been found to be functionally expressed in different types of cancer cells, and to regulate different aspects of tumor cell behavior (cell proliferation, apoptosis, migration, invasiveness etc.)^26^. Therefore, ICT could represent novel biomarkers and therapeutic targets of cancer^22^,^27^,^25^,^28^,^26^. Overexpression of ICT and Na^+^/H^+^ exchanger are now considered hallmark of cells undergoing tumorigenesis, resulting in raised intracellular cation concentration and pH value, which stimulates tumorigenesis and rapid tumor growth and metastasis^29^,^30^,^31^,^32^. Na^+^ is the major cation increased significantly in tumor cells than nontumor cells^10^,^11^. Therefore, detection of Na^+^ MCV is theoretically promising in intraoperative tumor diagnosis.

Based on the present study, Na^+^ MCV detector might have potential in complementing frozen biopsy from cell morphology to cell metabolism with several advantages: (1) short detection time. The average detection time is five to ten seconds for one site and two to three minutes for one sample, which are significantly shorter than frozen examination. (2) Multiple sites detection. (3) Simple operation procedure. (4) Numerical output of the detection results serves as the basis of clinical diagnosis index and standards.

In conclusion, our results showed that Na^+^ MCV was significantly upregulated in malignant tumor compared to benign tumor or normal tissue, and to the paired paracancerous tissue or surgical margin tissue. Accordingly, Na^+^ detector, being fast, convenient and effective, may have diagnostic potential in occupying lesions and serve as complementary methods to frozen examination during operation. Studies with larger populations enrolled are needed to assess the validation and optimal threshold of Na^+^ detector, especially in specific tissue, in near future.

## Materials and Methods

### Na-1 Na^+^ detector

**Figure 3:**
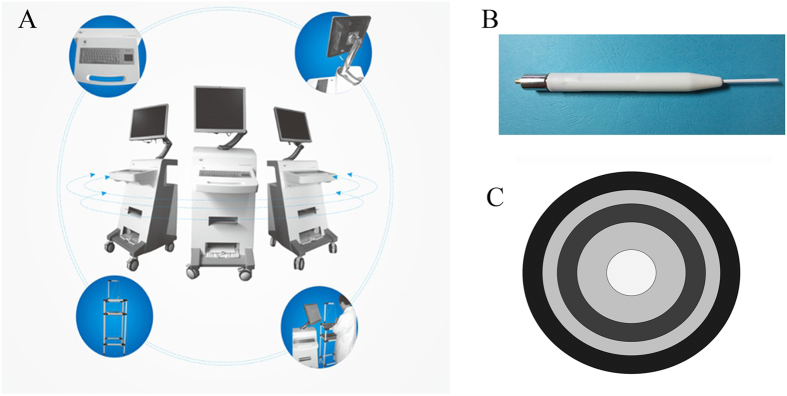
Structure and accessories of Na-1 Na^+^ detector. (**A**) The apparatus is composed of four fundamental parts: probe, host (sensor and processor), mornitor and operating table. (**B**) Overall structure of the probe. (**C**) Diagram of transversal surface of the probe.

Na-1 Na^+^ detector has been manufactured under the collaboration between Jiangsu Kunshan Bokang Medical Technology Co. Ltd. and our laboratory (Registration NO. Su SFDA (quasi) word 2012 NO. 2400987th). The detector is mainly composed of four parts: the probes, the host (sensor, processor), display and operation table (Fig. 3A). The probe is composed of a cylindrical sensor and insulated shell. The cylindrical sensor is the main function unit, the diameter of which is only 2 mm (Fig. 3B). The sensor is functionally similar to a closed micro-electronic chemical battery, consisting of the conductor, inner conductor, inner insulator, outer conductor and outer insulator (Fig. 3C).

When the sensor electrode (2 mm) contacts with tissue cells, potential difference will be generated between intracellular cation and electronic chemical batteries and loop current will be formed from the sensor and tissues. After processing of the detected signal by detector host, the MCV will be showed with continuous curves on the display. The MCV is positively associated with intracellular cation concentration.

## Materials

### Tissue collection

The specimens from 139 patients undergoing tumor resection were collected in the operation theatre from August 2013 to January 2014 in the First Affiliated Hospital of Medical College, Xi’an Jiaotong University. Amongst, there were 60 males and 79 females, with an average age of 53 years (25–78 years). All the fresh tissues were collected in the operation room within 30 min after isolation. In accordance with the pathologically specimen collection, the mass, nodules and lumps were cut open. Two or more sections were incised for larger tissues specimen (more than 5 cm × 3 cm). Different parts of the specimen were carefully observed for color, hardness, necrosis and calcification. The study was approved by the ethics committee of the First Affiliated Hospital, Xi’an Jiaotong University, the rules of which comply with the Treaty of Helsinki. Informed written consent for intraoperative detection of the tissue and use of the patient’s clinical data were requested from the patients or their relatives before surgery.

### Tissue cleaning

The fresh tissue specimen were washed with distilled water and then dried before test. Small piece of tissues were then immersed in distilled water and washed in a cleaning cup more than twice. The cleaned samples were covered with blotting paper, and then dried without water stains.

## Methods

### The methods were carried out in accordance with the approved guidelines

#### Pathological definition of the tumors

**Figure 4:**
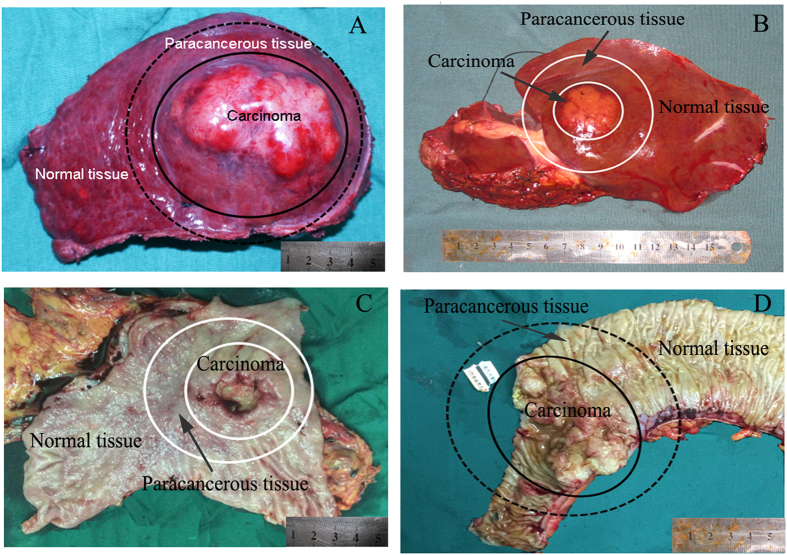
Representative images showing carcinoma, paracancerous tissue and paired normal tissue in liver (**A**,**B**), stomach (**C**) and colon (**D**). Paracancerous tissue was defined as the tissue less than 2 cm away from the tumor edge. Paired normal tissue was defined as the tissue more than 2 cm away from the tumor edge.

All the tumors and normal tissues were dissected from solid organs, which were firstly judged by the surgeons by naked eyes, and then confirmed by pathological examinations. Malignant tumor is defined as a neoplasm with invasive growth, no clear boundary with normal tissue, no or incomplete capsule formation, abnormal morphology changes with the normal tissue, poor differentiation with severe atypia, visible pathological mitoses, and mostly with necrosis, hemorrhage, or ulceration. The benign or borderline tumors were found expansive growth with intact capsule, clear boundary with normal tissue, similar tissue structure with the normal tissue, well cell differentiation with no or mild atypia, no or rare mitotic figures with no pathological mitotic figure. The 47 benign or borderline tumors included one hepatic adenoma, two pancreatic solid pseudopaillary neoplasm, one gallbladder adenomyoma, three gastic stromal tumor, 17 thyroid adenoma, five breast fibroadenoma, one lung tuberculoma, one renal hamartoma, two adrenal adenoma, one uterine cervix papilloma, five uterus myoma, one ovary cyst, and seven inflammatory nodules of lymph nodes (Table 1). Paracancerous tissue was defined as the tissue less than 2 cm away from the tumor edge. Paired normal tissue was defined as the tissue more than 2 cm away from the tumor edge (representative images, see Fig. 4). The final diagnosis of the tissue was confirmed by pathological examination. The diagnosis by Na^+^ detector was then evaluated based on the final pathological diagnosis.

#### Probe surface treatment

The probe was used for detection of MCV of cells. Before each test, the probe would be routinely cleaned with isopropanol and polished with sand plate on its surgace, since the surface material of the probe was susceptible to oxidation and electrochemical reaction.

#### Detection procedure

The detection sites included mass substance, paracancerous tissue, normal tissue and surrounding suspicious lymph nodes. The probe was tightly contact with the tissues. In the detection procedure on multiple sites, preliminary judgment of the specimen section (the same samples for pathological examination) including colors, density and quality was critical for the choice of stable detection sites. Generally, the sites showing high value and stable curves were selected as the reliable data for inspection report.

#### Data explanation

The MCV was plotted as curves extension with time on display. The vertical axis was the detected current value, ranging from 0 to 100 μA. The horizontal axis was the time axis. Stable data curve above the warning line (30 μA) was positive data signifying malignancy of tissues. The MCV for each tissue sites were documented.

#### Statistical analysis

Data were expressed as mean ± standard deviation for numerical variables, and percentages for nominal variables. Student’s t tests were used to compare numerical variables, and the chi-square test or Fisher’s exact test was applied to test differences in nominal variables between the groups. The diagnosis value of the Na^+^ detector was evaluated by area under receiver operating characteristic (ROC) curve. Statistical analysis was carried out using IBM^®^ SPSS^®^ Statistics^®^ version 21 for Windows (Chicago, Illinois). *p* < 0.05 was considered statistically significant.

## Additional Information

**How to cite this article**: Zhang, X.-F. *et al.* Na^+^ Micro-Current Value Detection as a New Modality for Identification of Benign and Malignant Disease in Surgery. *Sci. Rep.* **6**, 24937; doi: 10.1038/srep24937 (2016).

## Figures and Tables

**Table 1:** Distribution of the tissues detected in different organs.

Tissues origin	Tissue numbers	Malignant	Benign or borderline	Paracancerous tissue	Normal tissue	Surgical margin
Liver	19	7	1	3	4	4
Pancreas	26	9	2	4	7	4
Gallbladder/bile duct	39	10	1	9	11	8
Duodenal papilla	14	4	0	4	4	2
Gastrointestinal tract	76	18	3	18	19	18
Esophagus	18	5	0	4	5	4
Thyroid	40	8	17	0	15	0
Nose and throat	13	5	0	2	5	1
Breasts	21	10	5	0	6	0
Lungs	8	3	1	1	2	1
Kidneys	28	14	1	7	6	0
Adrenal gland	3	0	2	0	1	0
Ureters	3	1	0	1	1	0
Bladder	2	1	0	0	1	0
Testes tumor	2	1	0	0	1	0
Endometrium	9	3	0	3	3	0
Uterine cervix	11	3	1	3	4	0
Uterus myoma	6	0	5	0	1	0
Ovary	1	0	1	0	0	0
Lymph nodes	7	0	7	0	0	0
In total	346	102	47	59	96	42

**Table 2:** Comparison of the micro current value in malignant, benign or borderline and normal tissues.

Groups	Tissue type	Tissue number	Micro current value (μA)
1	Malignant tumor	102	33.3 ± 8.9
2	Benign or borderline tissue	47	24.4 ± 8.6
3	Normal tissue	96	14.0 ± 4.0

*p*(1 vs. 2) < 0.001; *p*(2 vs. 3) < 0.001.

**Table 3:** Comparsion of the micro current value in malignant tumor and paracancerous tissues.

Groups	Tissue type	Tissue number	Micro current value (μA)
1	Malignant tumor	102	33.3 ± 8.9
2	Paracancerous tissue	59	18.9 ± 4.1
3	Normal tissue	76	14.2 ± 4.0
4	Surgical margin tissue	41	15.2 ± 3.3

*p*(1 vs. 2, 3 or 4) < 0.001; *p*(2 vs. 3) < 0.001; *p*(3, 4) > 0.05.

**Table 4:** Na^+^ micro current value in different tissues of the organs and systems.

Tissues	Liver		Pancreas		Gall bladder & Bile duct		Gastrointestinal tract		Esophagus		Thyroid		Nose & throat		Breasts		Uterus tissue		Urinary system	
	*n*	MCV (μA)	*n*	MCV (μA)	*n*	MCV (μA)	*n*	MCV (μA)	*n*	MCV (μA)	*n*	MCV (μA)	n	MCV(μA)	n	MCV (μA)	n	MCV (μA)	n	MCV (μA)
Malignant tumor	7	31.1 ± 12.3*	9	39.6 ± 10.5*	10	36.7 ± 2.8*	22	36.2 ± 5.8*	5	39.0 ± 2.2*	8	33.0 ± 8.9*	5	33.8 ± 6.1*	10	35.5 ± 5.2*	6	27.2 ± 7.6*	16	21.1 ± 4.7
Paracancerous tissue	3	16.7 ± 5.1	4	17.8 ± 3.4	9	19.7 ± 3.0	22	19.6 ± 3.2	4	16.8 ± 2.2	17	22.5 ± 7.3 #	2	17.0 ± 7.1	5	22.4 ± 8.4 #	6	18.8 ± 5.6	8	20.0 ± 3.7
Surgical margin tissue	4	17.5 ± 2.1	4	16.3 ± 4.1	8	18.3 ± 1.7	20	15.9 ± 3.1	4	13.8 ± 3.1	–	–	1	7.0	–		5	24.8 ± 4.0 &	–	–
Normal tissue	4	15.3 ± 3.8	7	14.0 ± 3.8	11	15.5 ± 2.0	23	14.8±3.4	5	10.8 ± 3.1	15	14.9 ± 3.4	5	16.0 ± 3.1	6	7.0 ± 2.0	8	13.9 ± 4.5	8	14.0 ± 5.3

MCV, micro current value. *p < 0.05, compared between malignant tumor and paracancerous tissue, benign tumor, surgical margin tissue or normal tissue.

^#^benign tumors, ^&^ uterine myoma.

**Table 5:** Comparison between the pathological examination and Na^+^ micro current value detector.

	Pathological examination		
	Malignant tumor		Benign/borderline tumor
≥30 μA	70 (53.0%)		8 (6.2%)
<30 μA	14 (10.8%)		38 (29.2%)
Coincidence rate		83.1%	
Sensitivity		83.3%	
Specificity		82.6%	
Positive predictive value		89.7%	
Negative predictive value		73.1%	
The area under ROC curve		0.842	
